# Measuring the costs of outreach motivational interviewing for smoking cessation and relapse prevention among low-income pregnant women

**DOI:** 10.1186/1471-2393-9-46

**Published:** 2009-09-23

**Authors:** Jennifer Prah Ruger, Karen M Emmons, Margaret H Kearney, Milton C Weinstein

**Affiliations:** 1 Yale University Schools of Medicine and Public Health, New Haven, CT, USA; 2 Division of Community-Based Research, Dana Farber Cancer Institute, Harvard Medical School and Department of Society, Human Development and Health, Harvard School of Public Health, Cambridge, MA, USA; 3 School of Nursing, University of Rochester Medical Center, Rochester, NY, USA; 4 Departments of Health Policy and Management and Biostatistics, Harvard School of Public Health and Department of Medicine, Harvard Medical School, Cambridge, MA, USA

## Abstract

**Background:**

Economic theory provides the philosophical foundation for valuing costs in judging medical and public health interventions. When evaluating smoking cessation interventions, accurate data on costs are essential for understanding resource consumption. Smoking cessation interventions, for which prior data on resource costs are typically not available, present special challenges. We develop a micro-costing methodology for estimating the real resource costs of outreach motivational interviewing (MI) for smoking cessation and relapse prevention among low-income pregnant women and report results from a randomized controlled trial (RCT) employing the methodology. Methodological standards in cost analysis are necessary for comparison and uniformity in analysis across interventions. Estimating the costs of outreach programs is critical for understanding the economics of reaching underserved and hard-to-reach populations.

**Methods:**

Randomized controlled trial (1997-2000) collecting primary cost data for intervention. A sample of 302 low-income pregnant women was recruited from multiple obstetrical sites in the Boston metropolitan area. MI delivered by outreach health nurses vs. usual care (UC), with economic costs as the main outcome measures.

**Results:**

The total cost of the MI intervention for 156 participants was $48,672 or $312 per participant. The total cost of $311.8 per participant for the MI intervention compared with a cost of $4.82 per participant for usual care, a difference of $307 ([CI], $289.2 to $322.8). The total fixed costs of the MI were $3,930 and the total variable costs of the MI were $44,710. The total expected program costs for delivering MI to 500 participants would be 147,430, assuming no economies of scale in program delivery. The main cost components of outreach MI were intervention delivery, travel time, scheduling, and training.

**Conclusion:**

Grounded in economic theory, this methodology systematically identifies and measures resource utilization, using a process tracking system and calculates both component-specific and total costs of outreach MI. The methodology could help improve collection of accurate data on costs and estimates of the real resource costs of interventions alongside clinical trials and improve the validity and reliability of estimates of resource costs for interventions targeted at underserved and hard-to-reach populations.

## Background

Economic theory provides the philosophical foundation for valuing costs in judging medical and public health interventions. Resources used in, and saved as a result of, medical and public health programs must be measured in terms of their monetary costs to understand the overall implications of such interventions. Under economic theory, the opportunity cost of a resource - or its value in its next best or alternative use - is the real cost of that resource to society. Under ideal circumstances (e.g., perfect competition, absence of market failures such as externalities and public goods and absence of market distortions such as insurance) the price of a resource can be taken as its opportunity cost. However, medical and public health programs rarely operate under ideal market conditions. Thus, we cannot always rely solely on prices to value all resources in the economic evaluation of medical and public health interventions, although prices are almost always used for cost estimates. Rather, we need a systematic methodology for measuring costs grounded in the theory and process of identifying, estimating and valuing resource costs. This method can be described as micro-costing.

Despite recent work on economic evaluation in clinical trials [1], which focuses, for example, on econometric techniques for the analysis of cost distributions, little, if any, research has been conducted on micro-costing in health and medicine, particularly in conjunction with randomized controlled trials (RCT). Cost analyses in many areas of public health and medicine, including smoking cessation, have not typically been conducted uniformly and standardized so as to compare cost estimates across studies. Many have not included future costs, patient time and travel, or set-up and implementation costs. Incomplete and inadequate cost analyses can have a significant effect on final cost analyses (especially where real costs are underestimated), and on cost-effectiveness and cost-utility analyses. Cost analyses require standardized methodology as recommended by the Panel on Cost-Effectiveness in Health and Medicine [2] (hereinafter, the Panel). The lack of standardized or uniform methods in estimating costs is problematic because it limits our ability to determine which resources allocated to medical and public health programs are used more equitably and efficiently. This article aims to address these gaps in the literature.

Currently available economic evaluations of behavioral counseling services for smoking cessation provide limited evidence on costs to determine optimal resource allocation strategies. With some notable exceptions, few studies have been performed in accordance with U.S. Panel on Cost-Effectiveness in Health and Medicine recommendations grounded in economic theory. While such limitations hinder comparisons among programs, they also represent an opportunity for future analyses to better adhere to sound and consistent methodologies. A lack of economic studies is common in public health and medicine, however, especially in the area of prevention services [3]. Despite these limitations, efforts to sort out the finer methodological challenges in the cost elements of economic evaluation will eventually lead to more uniformity in reporting. For example, efforts to enumerate cost categories will improve the generalizability and comparability of the results of economic evaluations. In combination with established standards, such efforts will help in the development of an analytic framework for making future studies more comparable. Moreover, efforts to design and conduct economic evaluations of drug abuse treatment, smoking cessation, and relapse prevention, would ideally be planned prospectively and apply standardized methods.

Smoking imposes a significant economic burden on society, and smoking cessation can improve both the quantity and quality of life at low costs [4,5]. When evaluating smoking cessation interventions, accurate data on costs are essential for understanding resource consumption. Smoking cessation interventions, for which prior data on resource costs typically are not available, present challenges. Outreach programs, in particular, present special challenges because resources are concentrated in the field; yet, estimating the costs of outreach programs is critical for understanding the economics of targeting underserved and hard-to-reach populations. Therefore, we developed a methodology for collecting and providing cost data for outreach smoking cessation and relapse prevention programs such as motivational interviewing (MI). MI is a participant-tailored technique, which explores perceptions and concerns about smoking, clarifies conflicting motivations, focuses on participants' social contexts, and provides support and skills training. It aims to reduce household levels of nicotine, increase readiness to quit, and lower relapse rates. MI was originally developed in the specialist setting for use with chronic alcoholics and, over the past several years, has increasingly been adapted for use in public health settings [6,7]. Although MI is increasingly being adapted and used in addiction research, few, if any, studies have measured the costs of delivering this technique in outreach settings.

In a randomized controlled trial (1997-2000), we compared MI on an outreach basis (N = 156) with Usual Care (UC) (N = 146) among low-income pregnant women in Boston. During an average of three home visits, a nurse delivered the MI intervention and built a relationship with the participant. The MI components were tailored to each participant's stage of readiness.

Because this program consumed a considerable amount of professional time (e.g., for intervention training, preparation, scheduling, and delivery, and for travel to and from the intervention delivery site), it was important to precisely estimate its true costs. Therefore, we used micro-costing, which directly enumerates and subsequently values each input consumed for each particular participant. This approach's level of detail is especially important when personnel time or training account for a major portion of an intervention's costs. Because it is difficult to retrospectively create records of personnel time expended during a behavioral intervention, these data must be tracked during program delivery. For outreach programs, travel time and mileage must also be collected prospectively. To capture these details, we developed a process tracking system.

When assessing the full costs of smoking cessation and relapse prevention programs, it is also important to account for potential future resource use or savings that might result from the intervention. Published estimates of net lifetime additional medical costs for smokers [8-10] and net smoking-attributable medical costs for neonatal intensive care, chronic medical conditions, and acute conditions during the first year of life [11] provide evidence for the significance of these costs. Gross-costing (e.g., cost estimate of typical costs for a good or service) of clinical events prevented is more suitable for this purpose, but its estimates should be included in sensitivity analyses to assess their importance to the overall analysis [2].

When the Panel on Cost-Effectiveness in Health and Medicine recommended micro-costing in the late 1990s [2], it suggested breaking the production and delivery of an intervention into discrete work-steps, which could be analyzed separately; however, it gave few guidelines on how to implement a micro-costing study, in general, and no guidelines or procedures on how to do so in particular studies. For each step, all inputs, including personnel and patient time and supplies and equipment, are inventoried and measured. The costs of each step are summed to determine the intervention's total cost [2]. The straightforward micro-costing methodology developed here was grounded in economic theory and undergirded by the Panel's recommendations, which we modified and applied to an outreach MI program. As an input to cost-effectiveness analyses (CEA) and cost-benefit analyses (CBA), this methodology could enable benefits to be weighed against costs to facilitate rational allocation decisions. Cost estimation and analysis need not, however, be confined to use in CEA or CBA, but may be relevant as a stand-alone measure of economic impact, as an element of cost-minimization analysis (CMA) and as a component of a larger theory of health and social justice [12].

## Methods

### Recruitment, design, and sample of randomized controlled trial

Potential study participants were recruited from a large number of community-based health care practices and community health care centers that provided prenatal care. To be eligible, women had to be less than 28 weeks pregnant, speak English or Spanish, and be either current smokers or recent quitters (e.g., quit during previous three months). They also could not be in drug addiction treatment. During routine prenatal visits, health care providers described the study and assessed women's interest in participating. Study personnel then received contact information and called interested women for further explanation of the study. Trained research assistants visited the women in their homes to answer questions, obtain informed consent, conduct the baseline survey, and complete other study assessments. The health care providers referred 549 women to the study. Of those, 65 did not meet eligibility requirements, and 68 could not be located. Of the remaining 416 women, 114 refused participation. The final sample at baseline was 302 pregnant women (73% of known eligible women).

After the 302 women gave their consent and completed the baseline assessment, they were randomly assigned to either UC or MI. Follow-up assessments were conducted 10 weeks after baseline (prenatal assessment) and 4 to 6 months after the baby's birth (postnatal assessment). At each assessment visit, passive sampling dosimeters were placed in the home (kitchen and living room) for 7 days to assess air nicotine concentrations. Feedback about nicotine levels was provided as part of the MI intervention.

### Intervention conditions

#### Usual care

After UC participants completed the baseline survey, a letter was sent to their prenatal care providers indicating the patient's study participation and recommending that the provider discuss the patient's smoking with her. A description of the study, a tip sheet for providers based on the Agency for Healthcare Research and Quality (AHRQ) guidelines [13] and a pamphlet describing ways of dealing with nicotine withdrawal symptoms were included with the letter. Participants in this condition received their usual prenatal care from their providers.

#### Motivational interviewing

In addition to their usual care, MI participants received monthly visits from a Public Health Nurse until one month post-partum. The intervention was designed to address social contextual factors that might influence responses to the smoking intervention. The nurse was available to help with all aspects of pregnancy, including prenatal education, gaining needed social services, smoking cessation and secondhand smoke reduction.

The intervention was based on motivational interviewing [14], which emphasizes the woman's choice, personal responsibility for change, and enhancement of self-efficacy. The MI component was designed to increase motivation and self-efficacy for smoking cessation and to provide support and skills-training. Thus, the sessions explored the participants' perceptions and concerns about smoking, increasing their awareness of the pros and cons of smoking and clarifying conflicting motivations governing decision-making. The goals of the MI session were: (1) to provide information about the impact of smoking on the mother and fetus and the impact of secondhand smoke on newborns; (2) to help participants re-evaluate their smoking behavior; (3) to increase perceived self-efficacy for the ability to quit smoking; (4) to educate the participants about mechanisms for reducing secondhand smoke exposure and to set goals for changing smoking habits; and (5) to provide feedback about the impact of changes on household nicotine levels. These MI components were tailored to each participant's stage of readiness.

The first counseling session focused on increasing motivation, in part by addressing ambivalence associated with the decision to quit smoking. The nurses began the counseling session by presenting results from household nicotine assessments. If the woman was interested, the nurse discussed ways to either reduce the nicotine levels in the home or, if levels were low, ways to prevent them from increasing. Smoking cessation was discussed with women who were interested. Additionally, the nurses worked with the participants on setting goals in other areas of their lives (e.g., employment, education, parenting skills, social service needs). When appropriate, the relationship between these other goals and smoking was explored. The first counseling session placed a strong emphasis on goal setting. Progress toward those goals was the focus of the subsequent intervention visits.

### Micro-costing methodology

The theory and process of valuing costs through a micro-costing methodology rests on a three-step approach: identification, measurement, and valuation of resources used. While the Panel recommended following this theory and process, it provided no guidelines on implementing a micro-costing study, in general, and no procedures on how to do so in specific studies. Because micro-costing uses primary data on the exact number and type of resources consumed by each participant, it is most accurate when it tracks resource consumption as it occurs, thereby enhancing the validity and reliability of cost estimates. Once resource utilization is measured, the quantity of each type of resource consumed is multiplied by unit costs, and the results are summed to obtain total component-specific costs and overall cost. Total and component-specific costs can then be divided by the number of participants to determine expected costs per participant.

The Panel also recommended that future costs and savings attributable to an intervention be included in cost analyses from the societal perspective, in addition to micro-costing of intervention costs. Many interventions, especially preventive ones, for example, can result in future savings that offset initial costs. Analyses performed from the perspective of a provider or payer would include only those costs and savings that they bear or realize. Thus, in this micro-costing study, we include the patient's costs as well as the providers' costs and we conceptualize future savings that could offset initial costs. We conclude with sensitivity analyses of the robustness of these estimates to changes in key cost components.

### Step 1: Identifying resources used (component enumeration)

In step 1, it is important to thoroughly delineate all inputs that might be affected by the intervention so as to account for the cost of each component. This first step involves the identification of resources used in the "production process" of the intervention. Each "work step" in the production process is then described in conjunction with the relevant cost components. This step may also be called component enumeration, whereby each factor involved in the "production" of the intervention is identified and fixed costs (that do not vary with volume) are separated from variable costs (that do vary with volume). The Panel recommended that incremental costs (those required to produce an additional unit of service) rather than average costs (the total cost of an intervention divided by the units of services produced) be collected and analyzed.

As shown in Table 1, the costs for outreach MI can be represented by a three-way partition of cost categories: (1) set-up or implementation costs, including the costs of staff time and training materials; (2) time-dependent program costs (such as administrators' salaries or training costs for staff turnover), which are independent of the number of participants in a program, but incurred for as long as a program operates; and (3) variable program costs, which vary with the number of participants and include staff time spent interviewing participants, participant time, transportation, and consumables. Using this classification enables analysts to extrapolate to programs of different sizes.

**Table 1:** Three-way classification of resource utilization and costs

**Set-Up or Implementation Costs**

1. Training Costs
2. Marketing Costs

**Time-Dependent Program Costs**

1. Administrative Personnel
2. Durable Equipment
3. Program Space
4. Utilities

**Variable Program Costs**

1. Staff Time
2. Patient/Participant Time
3. Transportation
4. Consumables (e.g., materials)
5. Analysis of Nicotine Samples

Table 2 lists the cost components for outreach MI: (1) training costs (including time and travel for trainers and trainees, teaching materials, hand-outs and overheads, training manuals, refreshments); (2) personnel costs (scheduling, preparation, and delivery of intervention, travel time, distribution of intervention materials, collection of biochemical assessments included in the intervention); (3) travel costs (including mileage to and from intervention site); (4) patient/participant time during MI; (5) intervention materials (including printed materials); and (6) analysis of nicotine samples (used as a component of the intervention).

**Table 2:** Resource utilization categories and costs

**Resource Utilization Components**	**Measurable Units (Q)**	**Resource Valuation (P)**	**Cost per Unit of Consumption**
**Set-Up or Implementation Costs**			

Training Staff (Nurses & Supervisor)			

Trainer Time	Minutes	Hourly Wage Rate	TC*

Trainer Transportation	Miles	Cents per Mile	TC**

Trainee Time	Minutes	Salary + Fringe	TC*

Trainee Transportation	Miles	Cents per Mile	TC**

Training Manuals	Item	Production Costs	TC

Overheads and Hand-Outs	Item	Production Costs	TC

Refreshments	Item	Purchased Costs	TC

**Variable Program Costs**			

Staff Time (Nurses)			

Scheduling	Minutes	Salary + Fringe	TC*

Preparation	Minutes	Salary + Fringe	TC*

Intervention Delivery	Minutes	Salary + Fringe	TC*

Travel	Minutes	Salary + Fringe	TC*

Patient/Participant Time	Minutes	Hourly Wage Rate	TC*

Transportation	Miles	Cents per Mile	TC**

Self-Help Materials	Item	Production Costs	TC

Analysis of Nicotine Samples	Samples	Lab Costs	TC

*(TC = P*Q) product of price (P; salary & fringe or wage rate costs per minute) and quantity (Q; average minutes per person). TC is Total Costs.

**(TC = P*Q) product of price (P; Cents per Mile == $0.25) and quantity (Q; average roundtrip miles)

To determine the amount of personnel time involved in the intervention, we had to describe the work-steps involved. They included scheduling and follow-up, preparation, travel, and intervention delivery (counseling sessions). The process tracking form served as a generic flow chart for these steps.

We did not include the clinics' overhead costs (e.g., laundry, heating expenses) because they were similar in the intervention (MI) and control (UC) groups and as an outreach program, whereby the set-up and delivery of the intervention occurred "off-site," the similar overhead costs would likely be minimal. Because the participants were interviewed at home, clinic and hospital operating expenses (facilities costs) were not relevant. Neither were productivity costs [2] (participant work time lost due to morbidity or mortality), though participant time spent receiving MI was included.

Extending the cost analysis to the societal perspective would include net resource costs: (1) the intervention costs described above; (2) cost savings for neonatal intensive care, chronic medical conditions, and acute conditions during the first year of life; and (3) cost savings for maternal health care (e. g., reductions in lifetime medical expenditures for cardiovascular and lung diseases).

### Step 2: Measuring resource use (the process tracking system)

To enhance the reliability and validity of our cost data, we measured and valued all inputs consumed as the program proceeded (as part of the RCT), rather than trying to reconstruct them retrospectively. To collect utilization data in the RCT, we developed a process tracking form for manually recording information on: (1) components delivered; (2) time spent with each participant to conduct the intervention or follow-up; (3) materials provided; and (4) travel times and distances. The costs collected were those necessary for reproducing the intervention in a non-research setting [2]. They included: (1) staff time spent to deliver the intervention; (2) cost of determining environmental nicotine levels (used in MI); (3) cost of training the nurses to deliver the intervention; and (4) cost of producing the self-help materials. The nurses tracked their resource consumption and time themselves, distinguishing intervention time from research and evaluation time. Thus, the process tracking form collected the number of minutes nurses spent contacting participants (scheduling) and preparing and delivering the intervention (from start to finish), documented the distance in miles to a participant's home, and listed the amounts of materials consumed (e.g., the number of pamphlets). It also had space for notes.

Time spent scheduling and contacting participants is typically an administrative cost, but we decided that nurses, rather than clerical staff, should have phone contact with participants before visiting their homes. The only additional support required was from a nurse supervisor, who was trained in delivering the MI intervention but spent little effort on the intervention itself.

Invoices for photocopying and other production expenses were filed by research administrators and used as the bases for cost estimates and to confirm quantities. Facilities costs (e.g., utilities and custodial services) were deemed similar in both groups, and therefore excluded. No new or existing equipment was required.

A significant category of costs was, however, transportation costs. Participants did not incur travel costs because they received the intervention at home, but nurses did and they logged their time spent during travel and the number of miles to and from participants' homes on the process tracking form. We did not assume "stacking" of travel (e.g., a nurse might visit 3-4 houses at one time in a given community), although a program like this could involve "stacked travel," with potentially less travel time for outreach nurses. While participants did not spend time traveling to and from the intervention, they did spend time participating in it. Nurses logged participant time spent in the intervention, again through the process tracking system.

For our outreach MI program, mean values for staff time were: (1) scheduling, 17 min; (2) preparation, 11 min; (3) intervention delivery, 66 min per session; and (4) travel to and from the delivery site, 40 min and 29 miles. Average participant time spent during MI was 66 minutes per session (Table 3).

**Table 3:** Variable program resource utilization

**Resource Components**	**Mean Units per Participant**	**95% Confidence Interval**
Staff Time		

Scheduling	17 minutes	(14.9 to 19.2) minutes

Preparation	11 minutes	(9.7 to 12.7) minutes

Intervention Delivery	66 minutes	(63.0 to 68.9) minutes

Travel	40 minutes	(37.1 to 43.1) minutes

Patient/Participant Time	66 minutes	(63.0 to 68.9) minutes

Transportation	29 miles	(25.7 to 31.5) miles

Materials	7 items	N/A

Analysis of Nicotine Samples	2 dosimeters	N/A

### Step 3: Valuing resources

The next step in micro-costing is to value the inputs to the intervention and then multiply those values by the units of resource utilization. This generates the total costs overall as well as the costs per component for implementing MI on an outreach basis.

### Resource costs

#### Cost of personnel time

By far the largest cost component for the MI intervention was personnel time, the primary personnel being nurses. We used the nurses' actual wages and fringe benefits (obtained from the clinics' payroll and accounting offices) to assign a monetary value to personnel time; thus, these rates would have to be adjusted for interventions outside the Boston area. The average nurse's salary plus benefits was $31.45 per hour (based on an annual salary of $50,741 in 1997 dollars plus ~24% for benefits).

The calculations for determining the cost per unit of staff time are shown in Table 2. Total personnel costs (TC_p _= P*Q) were the product of price (P; salary & fringe costs per minute) and quantity (Q; average minutes per participant).

### Participants' time

The monetary value of the time the participants spent receiving the MI was estimated by their hourly wage. For low-income women, we used the minimum wage in 1997 obtained from the Bureau of Labor Statistics (BLS) [15].

### Costs of material, supplies, and laboratory tests

Costs of purchasing consumables used in the intervention were obtained from research administrators. Costs of laboratory tests used to analyze nicotine samples were recorded, as was the cost of photocopying the self-help materials.

### Future cost savings

Future cost savings associated with smoking cessation and relapse prevention cannot be determined while a clinical trial is in progress and therefore must be gross-costed, using rough estimates from the literature and sensitivity analysis. These costs were assumed to be zero in the base case. Published estimates of net lifetime additional medical costs for smokers are $6,239 (discounted 1990 dollars) [8-10]. Published estimates that include net smoking-attributable medical costs for neonatal intensive care, chronic medical conditions, and acute conditions during the first year of life range from $1,024 to $1,228 [11] (in discounted 1996 dollars). Non-medical net attributable savings or costs that are associated with early death from smoking (e.g., social security, long-term care, etc.) are excluded. These costs are included in the overall cost analyses reported from the societal perspective, but are not reported in the following tables of direct program costs.

### Sensitivity analyses

Sensitivity analyses were conducted to analyze the variation in total costs according to uncertainties in key cost categories, such as intervention delivery, staff travel, and analysis of nicotine samples.

## Results

### Calculating total program costs and cost per participant

Table 4 shows the total cost and per participant cost for the interventions. All costs were reported in 1997 dollars and adjusted, when necessary, using the medical care component of the consumer price index from the BLS. The main cost components of outreach MI were intervention delivery, travel time, scheduling, and training.

**Table 4:** Direct program cost components of MI and UC

**Mean Cost per Participant**			
**Cost Components**	**MI**	**UC**	**Cost Difference (95% CI)**

**Set-Up or Implementation Costs**			

Training Staff ($3,930 for MI)	$25.2	$0	$25.2

**Variable Program Costs**			

Staff Time			

Scheduling	$26.5	$0	$26.5 ($23.7 to $29.3)

Preparation	$17.2	$0	$17.2 ($15.1 to $18.9)

Intervention Delivery	$103.0	$2.6	$100.4 ($95.7 to $104.8)

Travel	$62.4	$0	$62.4 ($56.8 to $66.3)

Patient/Participant Time	$17.0	$0.42	$16.6 ($15.8 to $18.2)

Transportation	$21.8	$0	$21.8 ($18.7 to $23.5)

Materials	$4.7	$1.8	$2.9

Analysis of Nicotine Samples	$34.0	$0	$34.0

**Total Variable Costs**	$286.6	$4.82	$281.8 ($264 to $298)

**Total Costs**	$311.8	$4.82	$307 ($289.2 to $322.8)

The total fixed costs of the MI were $3,930 and the total variable costs of the MI were $44,710. The total cost of the MI intervention for 156 participants was $48,672 or $312 per participant. The costs and costs per participant of the individual components were (totals may not add due to rounding):

### Set-up or implementation costs

• Eight-hour training session for the nurses and their supervisor = $3,930 or $25.2 per MI participant

### Variable program costs

• MI Scheduling - 17 minutes on average; @ $0.52/minute = $8.8 per intervention session or $26.5 per participant

• MI Preparation - 11 minutes on average; @ $0.52/minute = $5.7 per intervention session or $17.2 per participant

• MI Delivery - 66 minutes on average; @ $0.52/minute = $34.3 per session or $103 per participant

• Travel to and from the intervention delivery site - 40 minutes on average; @ $0.52/minute = $20.8 per visit or $62.4 per participant

• Participant time spent in MI - 66 minutes on average; @ a minimum wage of $5.15 per hour = $17.0 per participant

• Transportation to and from the MI delivery site for mileage (29 miles) @$0.25 per mile = $21.8 per participant

• Printed self-help materials (7 items for the MI group and 2 for UC group) = $4.7 (MI) per participant and $1.8 (UC) per participant, a difference of $2.90 per MI participant

• Cost of analyzing a nicotine sample - $17 per sample = $34 per participant (2 samples analyzed)

The total cost of $311.8 per participant for MI intervention compared with a cost of $4.82 per participant for usual care, a difference of $307 ([CI], $289.2 to $322.8) (Table 4). Using the expected fixed cost ($3,930) and variable cost per participant ($287) as a guide, one is then able to estimate the cost of providing MI to greater or lesser numbers of participants. For example, the total expected program costs for delivering MI to 500 participants (Fixed cost + 500 * Variable cost) would be $3,930 + 500 * 287 or $147,430. This assumes no economies of scale in delivering the program such that fixed costs will decrease in the long run.

### Sensitivity analyses

We conducted sensitivity analyses to examine the variation in our total cost estimates as a result of variation in key parameters. We were most interested in examining the effects of uncertainties in key cost categories for MI: intervention delivery, staff travel, and analysis of nicotine samples. As shown in Table 5, we varied the intervention delivery costs to a 25% decrease and increase, resulting in a range of total costs from $286 to $338. For staff travel, we reduced and increased the costs by 30% resulting in a range of total cost per person of $293 to $331. Finally, decreasing and increasing the costs of analyzing the nicotine samples by 35% resulted in a range of total costs per person of $300 to $324.

**Table 5:** Sensitivity analyses

**Resource**	**Uncertainties Examined**	**Total Cost per Participant**
Staff Time		

Intervention Delivery	+/- 25%	($286 to $338)

Travel	+/- 30%	($293 to $331)

Analysis of Nicotine Samples	+/- 35%	($300 to $324)

## Discussion

This study's aim was to develop a methodology and to measure and report the cost of outreach MI programs for smoking cessation and relapse prevention. The methodology offered here systematically applies the micro-costing framework recommended by the Panel for Cost-Effectiveness in Medicine in 1996 [2,16], but extends it through operationalizing costing techniques for an outreach program grounded in economic theory. It can be used, with minimal modifications, to estimate the costs of similar behavioral interventions, especially those that rely heavily on staff time and are outreach in nature. The process tracking system may be especially useful for collecting costs when training, delivery, scheduling, and travel are significant cost components.

Micro-costing using a process tracking system has many strengths and some limitations. The strengths include: 1) its systematic nature; 2) standardization across cost components; 3) reliability and validity resulting from cost collection during actual program delivery; and 4) ability to account for the most significant inputs (provider and participant time spent in training, scheduling, preparation, delivery, and travel). It is akin to detailed cost-accounting using the principles of industrial engineering and time-motion studies to estimate and compare actual production costs [17-22]. One limitation is the method's reliance on self-observation by providers, which may be prone to error. The alternatives--direct observation by a trained observer and patient flow analysis [23] --also have limitations, however, and each is susceptible to human error as well. Patient flow analysis is particularly problematic in that it places the burden for documentation and accuracy of reporting on the participant involved in the intervention. This method seems less desirable because it detracts from both the overall accuracy of data collection and intervention efficacy. Another limitation to micro-costing is the substantial burden it places on the research team and intervention personnel. Like the CCC's Cost Allocation Methodology [24], noted by the ADSS (Alcohol and Drug Services Study) Cost Study [25], micro-costing is a highly detailed and intensive method. Finally, limitations exist (e.g., differences in recruitment costs, scheduling issues, etc.) in translating cost estimates obtained through a randomized controlled trial to real world clinical practice.

There are other substance use treatment costing methods, such as the use of the Drug Abuse Treatment Cost Analysis Program (DATCAP) [26], and Substance Abuse Services Cost Analysis Program (SASCAP) [27]. However, these instruments tend to focus primarily on the institutional level in inpatient and outpatient treatment programs [28-32], aggregating and generalizing over the site of care delivery for typical costs of services. For the specific purposes of this study, they are less appropriate due to the use of integrated services such as behavioral counseling and due to the need to cost in detail these promising treatment services in conjunction with a randomized controlled trial. In this study, rather, we aimed to employ micro-costing to reflect the ideal of identification, measurement, and valuation of resources, guided by a theoretical framework that identifies all consequences of adopting different interventions.

Thus, despite the noted limitations, the detailed cost information collected by this methodology is essential for understanding the levels and types of resources necessary for effective implementation of smoking cessation and relapse prevention programs. As an input to cost-effectiveness analyses [33] and cost-benefit analyses, it could enable benefits to be weighed against costs to facilitate rational allocation decisions. Effective policy analysis and implementation of medical and public health programs relies on valid and reliable estimates of the costs and benefits of such programs. Micro-costing can help in providing these cost estimates. The methodology could help improve collection of accurate data on costs and estimates of the real resource costs of interventions alongside clinical trials and improve the validity and reliability of estimates of resource costs for interventions targeted at underserved and hard-to-reach populations.

## Competing interests

The authors declare that they have no competing interests.

## Authors' contributions

JR, MW, and KE conceived of the study concept and design, drafted the manuscript and supervised the study. All authors participated in data acquisition, analysis and interpretation of the data, and in critical revision of the manuscript for important intellectual content.

## Pre-publication history

The pre-publication history for this paper can be accessed here:


